# Donor-derived microbial engraftment and gut microbiota shifts associated with weight loss following fecal microbiota transplantation

**DOI:** 10.1128/aem.00120-25

**Published:** 2025-06-04

**Authors:** Yuting Ruan, Tongxi Zhu, Rui Yang, Fugui Su, Chiying An, Zhuping Hu, Xiaoli Li, Yue Li, Peizhao Chen, Xueni Shao, Junjie Qin, Hong Chen, Rongping Chen

**Affiliations:** 1 Department of Endocrinology and Metabolism, Zhujiang Hospital, Southern Medical University 70570, Guangzhou, China; 2 Southern Medical University 70570, Guangzhou, China; 3 Department of Endocrinology, Suixi Country People’s Hospital, Guangdong Medical University 12453, Guangzhou, China; 4 Department of Endocrinology, Shenzhen Hengsheng Hospital 733566 , Shenzhen, China; 5 Department of Endocrinology, Wengyuan Country People’s Hospital, Shaoguan, China; 6 Department of Endocrinology, The First People’s Hospital of Zhaoqing, Zhaoqing, China; 7 School of Life Science and Technology, School of Food Science and Engineering, Nutrition and Health Research Institute, Wuhan Polytechnic University 74615, Hubei, China; Universita degli Studi di Napoli Federico II, Portici, Italy

**Keywords:** gut microbiome, metagenome, obesity, fecal microbiota transplantation

## Abstract

Fecal microbiota transplantation (FMT) is a promising treatment for microbiota dysbiosis and may provide metabolic benefits for obesity. However, its mechanisms and variability in clinical outcomes remain poorly understood. This 12-week multicenter, single-arm study evaluated the efficacy of FMT for weight loss and explored the role of donor-derived microbial engraftment and functional shifts in mediating weight loss among overweight and obese individuals. Twenty-three participants (body mass index ≥24 kg/m²) without diabetes received three biweekly FMT sessions via a nasojejunal tube. Fecal samples from participants and donors were analyzed using metagenomic sequencing. By week 12, 52% of participants were classified as responders, achieving significant weight loss of ≥5% from baseline, with an average weight loss of 7.98 ± 2.69 kg (*P* < 0.001). In contrast, non-responders lost 2.90 ± 1.89 kg (*P* < 0.001). Responders exhibited a significantly higher proportion of donor-derived microbial strains post-FMT compared to non-responders (37.8% vs 15.2%, *P* = 0.020). Notably, key taxa, including *Phascolarctobacterium* (*P* = 0.034) and *Acidaminococcaceae* (*P* = 0.012), increased significantly in abundance in responders post-FMT, indicating successful microbial engraftment as a critical determinant of therapeutic success. These findings suggest that FMT is a viable intervention for weight loss in obese individuals. Successful donor-derived microbial engraftment strongly correlates with weight loss efficacy, highlighting the potential of microbiota-targeted therapies in obesity management and providing insights into the mechanisms underlying FMT outcomes.

**IMPORTANCE:**

Prior research indicates that fecal microbiota transplantation (FMT) is a promising treatment for diseases related to microbiota imbalance, potentially providing metabolic benefits for obesity. However, the specific role of donor-derived microbial engraftment in driving clinical efficacy has remained unclear. In this study, we evaluated the efficacy of FMT in promoting weight loss and explored the role of donor-derived bacterial strains in this process. Our findings demonstrate that the successful engraftment of specific donor-derived taxa, such as *Phascolarctobacterium* and *Acidaminococcaceae*, is strongly associated with significant weight loss. This highlights the critical interplay between donor microbiota and recipient gut environment. These findings underscore the potential of microbiota-targeted therapies as a novel strategy for obesity management.

**CLINICAL TRIALS:**

This study is registered with the Chinese Clinical Trial Registry as ChiCTR1900024760.

## INTRODUCTION

Obesity represents a significant global health challenge, contributing to a wide array of chronic conditions such as cardiovascular disease, hypertension, type 2 diabetes mellitus, and osteoarthritis (1). With its prevalence projected to affect over 4 billion individuals by 2035, effective therapeutic interventions have become a pressing priority (2). Emerging evidence highlights the pivotal role of gut microbiota in regulating physiological processes including food digestion (3), nutrient uptake and metabolism (4), and synthesis of vitamins and bile acids (5). Disruptions in the gut microbiome have been closely linked to obesity pathogenesis (6, 7). Fecal microbiota transplantation (FMT), a novel therapeutic approach involving the transfer of fecal microbiota from a healthy donor to recipients, has demonstrated the potential to restore microbial balance and improve outcomes in microbiota-associated conditions such as inflammatory bowel disease and infectious diseases (8). Recently, FMT has garnered attention for its potential to address obesity and metabolic syndrome. Notably, studies have shown that FMT can increase insulin sensitivity, with effects linked to enrichment of butyrate-producing microbiota in recipients (9). Similarly, it has been demonstrated that FMT from lean donors to obese patients with metabolic syndrome improves insulin sensitivity (10). Increased gut microbial diversity has also been observed post-FMT in obese individuals (9–12).

However, the impact of FMT on weight loss remains inconsistent across studies. Only one study has confirmed a correlation between specific bacterial strains and metabolic pathways with weight reduction following FMT (13). The precise constituents influencing outcomes remain largely elusive. A critical factor influencing the variability in FMT outcomes may be the composition of donor microbiota. For instance, studies have identified donor microbiomes with high microbial diversity and a favorable *Prevotella* to *Bacteroides* (*P*/*B*) ratio as key determinants of strain engraftment and metabolic improvement (11). Conversely, FMT from metabolically compromised obese donors has been shown to temporarily worsen insulin sensitivity, highlighting the importance of donor health and microbial composition (14).

In this study, we aim to explore the efficacy of FMT in promoting weight loss among overweight and obese individuals. Additionally, we seek to elucidate how donor and recipient microbiota characteristics influence therapeutic outcomes. By exploring the interplay between specific bacterial compositions and metabolic functions, our findings are intended to contribute to the development of targeted microbiota-based strategies for effective weight management strategies.

## RESULTS

### Clinical outcomes

**Fig 1:**
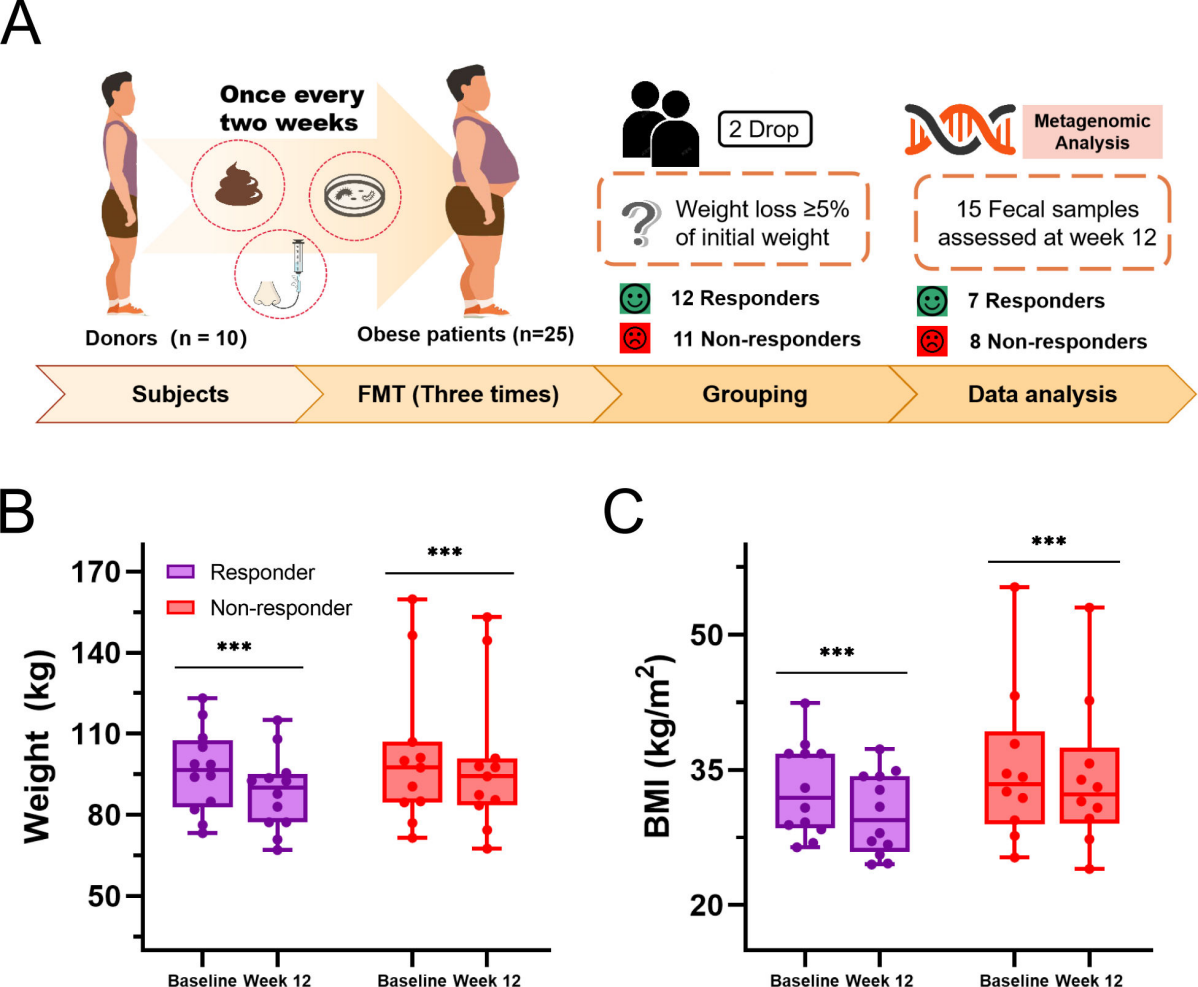
Study design and primary outcomes. (**A**) Study design illustrating the recruitment of 10 healthy donors and 25 obese participants. (B and C) Weight and BMI changes in participants at baseline and week 12. Data were analyzed using paired *t*-tests. ^*^P < 0.05, ^**^P < 0.01, and ^***^P < 0.001.

Of the 25 enrolled participants, 23 participants successfully received three FMT sessions, with 15 providing comprehensive metagenomic data at baseline and 12 weeks (Fig. 1A). Baseline characteristics of participants are shown in Table 1. The majority of participants were males (78%), with an average body mass index (BMI) of 33.81 kg/m^2^. Fecal samples used for FMT were donated by 10 healthy individuals with an average BMI of 19.62 kg/m^2^. Following three FMT sessions, 12 participants (52%) experienced a clinical response, defined as weight loss of ≥5% from their initial weight, while the remaining 11 participants did not meet this criterion. Individuals’ weight changes across the cohort are illustrated in Fig. 1B. A significant reduction in body weight was observed in both responders (6.3–9.7 kg; *P* < 0.001) and non-responders (1.6–4.2 kg; *P* < 0.001). Specifically, the average BMI in the responder group decreased by 3.00 kg/m^2^ (95% CI, 1.83–4.17), whereas the non-responder group showed a decrease of 1.23 kg/m^2^ (95% CI, 0.65–1.81) at 12 weeks (Fig. 1C). Importantly, donor-recipient matching analysis revealed similar weight loss outcomes among recipients who received FMT from the same donor, suggesting a potential influence of donor-driven effect on therapeutic efficacy (Fig. S1). However, no significant differences were observed in baseline characteristics between donors associated with responders and those associated with non-responders (Table S2).

**TABLE 1:** Baseline characteristics of recipients^*a*^

Characteristic	Data for:^*b*^			*P* ^*c*^
	Baseline	Responders (*n* = 12)	Non-responders (*n* = 11)	
Age	38.57 (11.31)	39.33 (9.99)	37.72 (13.04)	0.74
Male, no. (%)	18 (78%)	9 (75%)	9 (82%)	0.71
Body weight (kg)	99.02 (21.81)	96.39 (15.52)	101.88 (27.63)	0.36
Initial BMI (kg/m^2^)	33.81 (6.78)	32.85 (5.16)	34.85 (8.34)	0.49
Waist (cm)	106.29 (13.69)	106.89 (9.86)	105.69 (17.26)	0.85
Waist-hip ratio	0.97 (0.06)	0.96 (0.62)	0.98 (0.06)	0.47
BFR (%)	33.52 (6.29)	33.78 (6.46)	33.15 (6.47)	0.83
SBP (mmHg)	113.71 (18.16)	110.38 (20.60)	118.17 (14.88)	0.46
DBP (mmHg)	70.64 (16.01)	68.63 (20.22)	73.33 (8.89)	0.34
Fasting blood glucose (mmol/L)	6.35 (1.41)	6.33 (1.43)	6.38 (1.46)	0.93
Fasting insulin (mIU/L)	25.22 (16.42)	20.70 (11.18)	30.87 (20.70)	0.24
AST (IU/L)	26.59 (14.65)	23.30 (10.80)	30.25 (17.98)	0.72
ALT (IU/L)	45.74 (31.97)	43.15 (23.09)	48.32 (40.29)	0.40
Scr (umol/L)	72.26 (15.11)	69.76 (16.05)	74.76 (14.51)	0.62
BUN (mmol/L)	4.82 (1.36)	4.63 (1.48)	4.95 (1.33)	0.59
Uric acid (umol/L)	443.66 (129.18)	450.45 (133.31)	436.86 (131.73)	0.57
Cholesterol				
TG (mmol/L)	2.10 (1.22)	2.25 (1.54)	1.93 (0.76)	0.85
TC (mmol/L)	5.12 (0.97)	5.06 (1.14)	5.20 (0.79)	0.75
HDL-C (mmol/L)	1.08 (0.26)	1.05 (0.26)	1.12 (0.26)	0.55
LDL-C (mmol/L)	3.38 (0.77)	3.33 (0.92)	3.43 (0.61)	0.76

^*a*^ BFR, body fat rate; SBP, systolic blood pressure; DBP, diastolic blood pressure; TG, triglyceride; AST, aspartate aminotransferase; ALT, alanine aminotransferase; Scr, serum creatinine; BUN, blood urea nitrogen; TC, total cholesterol; HDL-C, high-density lipoprotein cholesterol; LDL-C, low-density lipoprotein cholesterol.

^*b*^ The data are shown as the mean (SD) or a number of subjects (%).

^*c*^ *P* value assessed by unpaired *t*-test for differences between responders and non-responders.

### Changes in fecal microbiota composition

**Fig 2:**
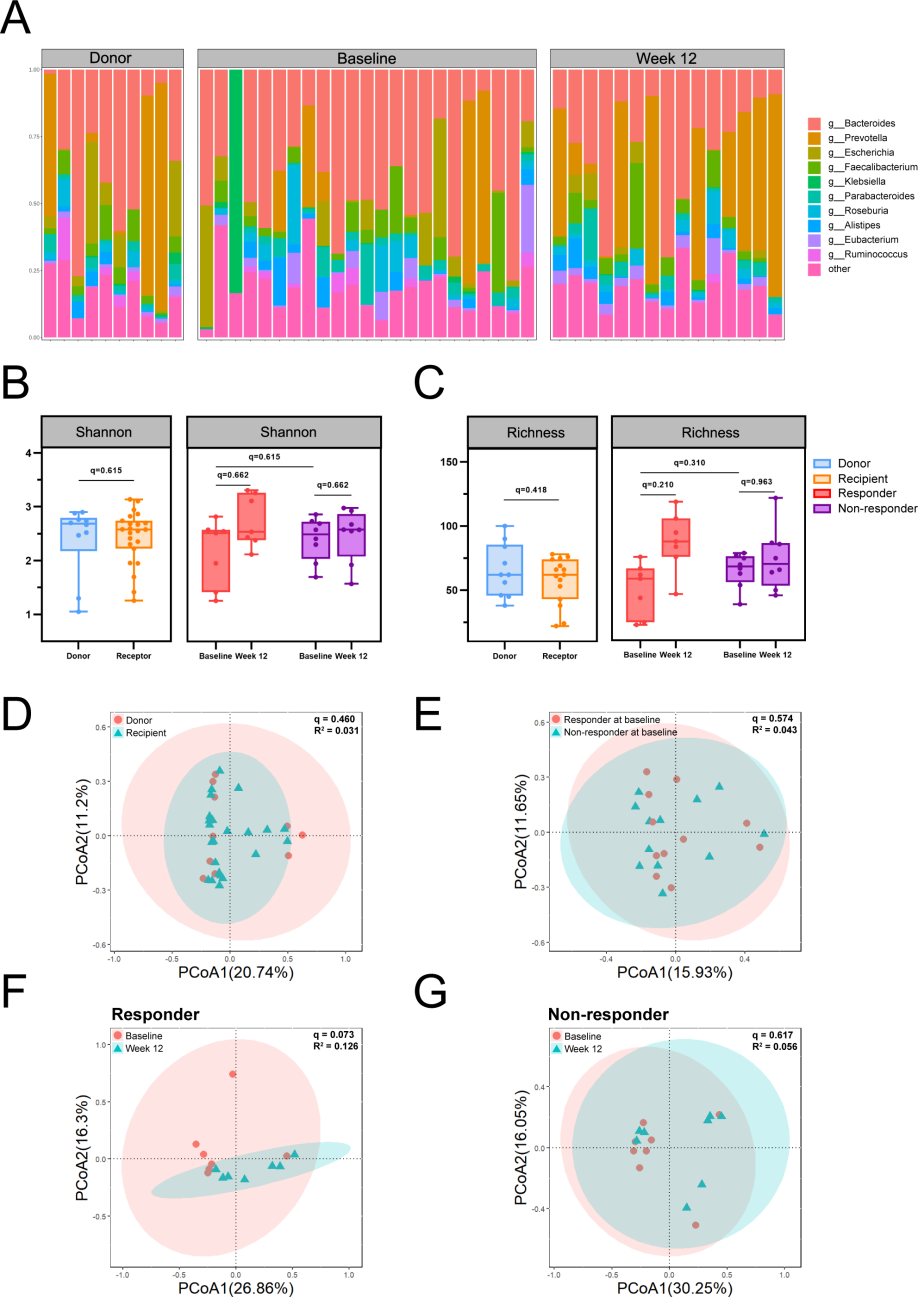
Gut microbiota assessment of donors and participants. (**A**) Microbiome composition of donors and participants at different time points. (B and C) Alpha diversity (Shannon and Richness) of the gut microbiome. Individual points represent separate samples. False discovery rate (FDR)-adjusted *P* values are indicated (^*^q < 0.05). (D–G) Principal coordinate analysis (PCoA) of Bray-Curtis dissimilarity data at the species level. FDR-adjusted *P* values are denoted (^*^q < 0.05).

To explore the relationship between microbial diversity and weight loss outcome, Shannon index and richness indices were analyzed across donors, recipients, responders, and non-responders (Fig. 2A through C). No significant differences were observed in alpha diversity between donors and recipients (Shannon: *q* = 0.615; Richness: *q* = 0.418) or between responders and non-responders at baseline and 12 weeks (Shannon: *q* = 0.662 and *q* = 0.662; Richness: *q* = 0.310 and *q* = 0.963). Additionally, recipients in the responder and non-responder groups showed no significant differences in baseline alpha diversity, indicating that initial microbial diversity in the gut was not predictive of clinical outcomes.

Beta diversity analysis revealed no significant differences in the overall microbial structure between donors and recipients at baseline (*q* = 0.460; Fig. 2D). Similarly, no significant differences in beta diversity were observed between responders and non-responders at baseline (*q* = 0.574; Fig. 2E). Among responders, a trend toward microbial compositional shifts was observed at week 12 compared to baseline, although this did not reach statistical significance after correction (*q* = 0.073; Fig. 2F). Conversely, non-responders exhibited minimal changes in microbial composition over time (*q* = 0.617; Fig. 2G).

### Donor microbiota diversity and functional pathways

**Fig 3:**
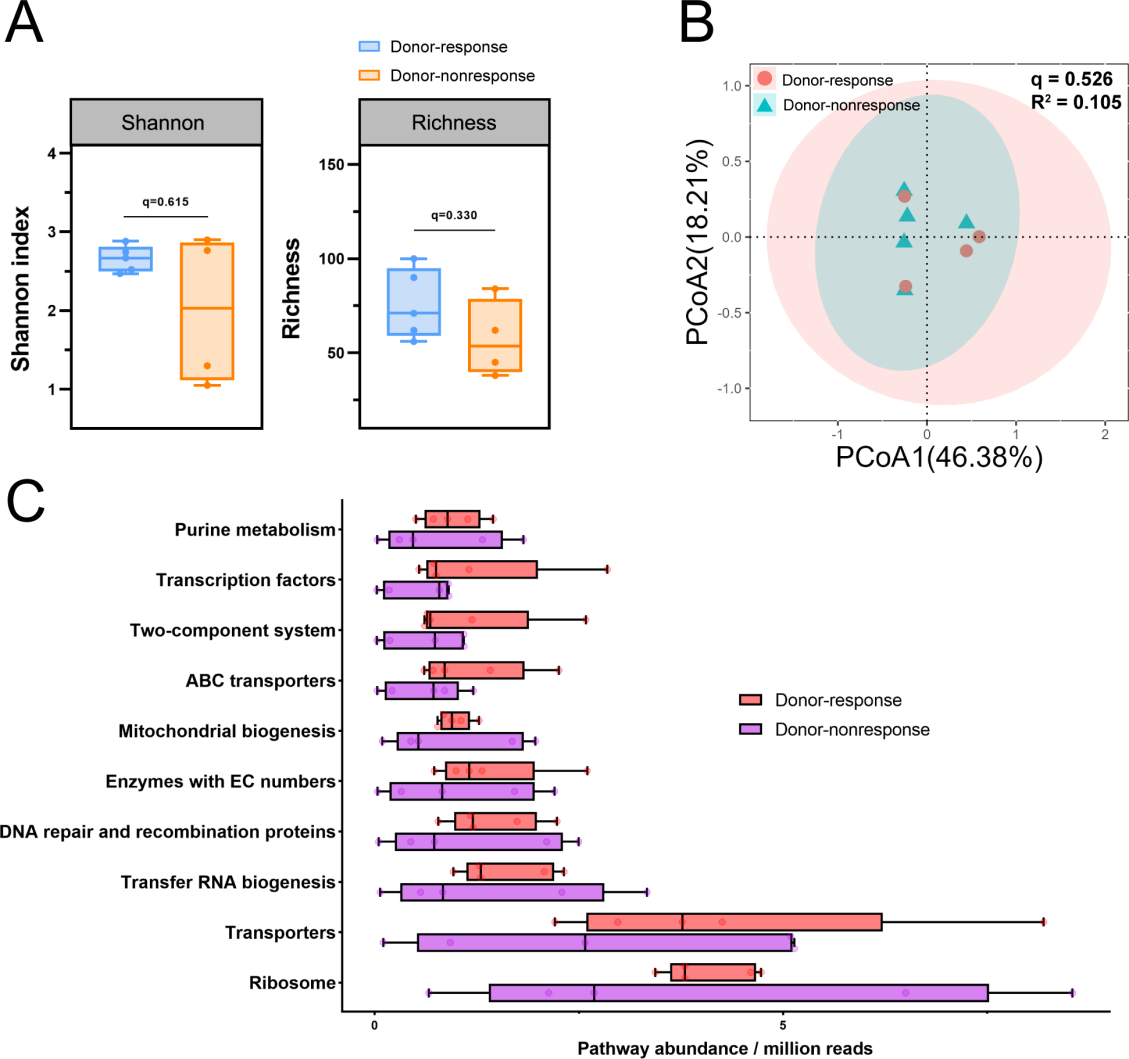
Gut microbiota assessment of donors paired with responders and non-responders. (**A**) Alpha diversity (Shannon and Richness) of the gut microbiome in donors paired with responders and non-responders. Individual points represent different samples. FDR-adjusted *P* values are denoted (^*^q < 0.05). (**B**) PCoA of Bray-Curtis dissimilarity data at the species level. FDR-adjusted *P* values are denoted (^*^q < 0.05). (**C**) Relative abundance of functional pathways in the gut microbiota of donors paired with responders and non-responders. No significant differences were observed, and the top 10 pathways were selected for presentation. Statistical analysis was conducted using independent-sample *t*-tests and Mann-Whitney *U* tests.

To determine the potential role of donor microbiota in influencing weight loss outcomes, microbial diversity, beta diversity, and functional pathways were compared between donor-response and donor-nonresponse groups. Table S3 provides detailed characteristics of the donor microbiomes. No significant differences were observed in the Shannon index (*q* = 0.615) or richness indices (*q* = 0.330) between donor groups (Fig. 3A). Beta diversity analysis similarly showed no clustering between donor-response and donor-nonresponse groups (*q* = 0.526; Fig. 3B). Subgroup analysis showed no distinct clustering for either donor-responder or donor-nonresponder pairs at baseline or post-FMT (Fig. S2). Functional pathway analysis further confirmed no significant differences in pathways such as purine metabolism and transcription factors (Fig. 3C). Together, these results suggest that donor microbiota composition may not fully explain the differential weight loss responses observed in recipients.

### Gut microbiome shifts toward donor in responders

**Fig 4:**
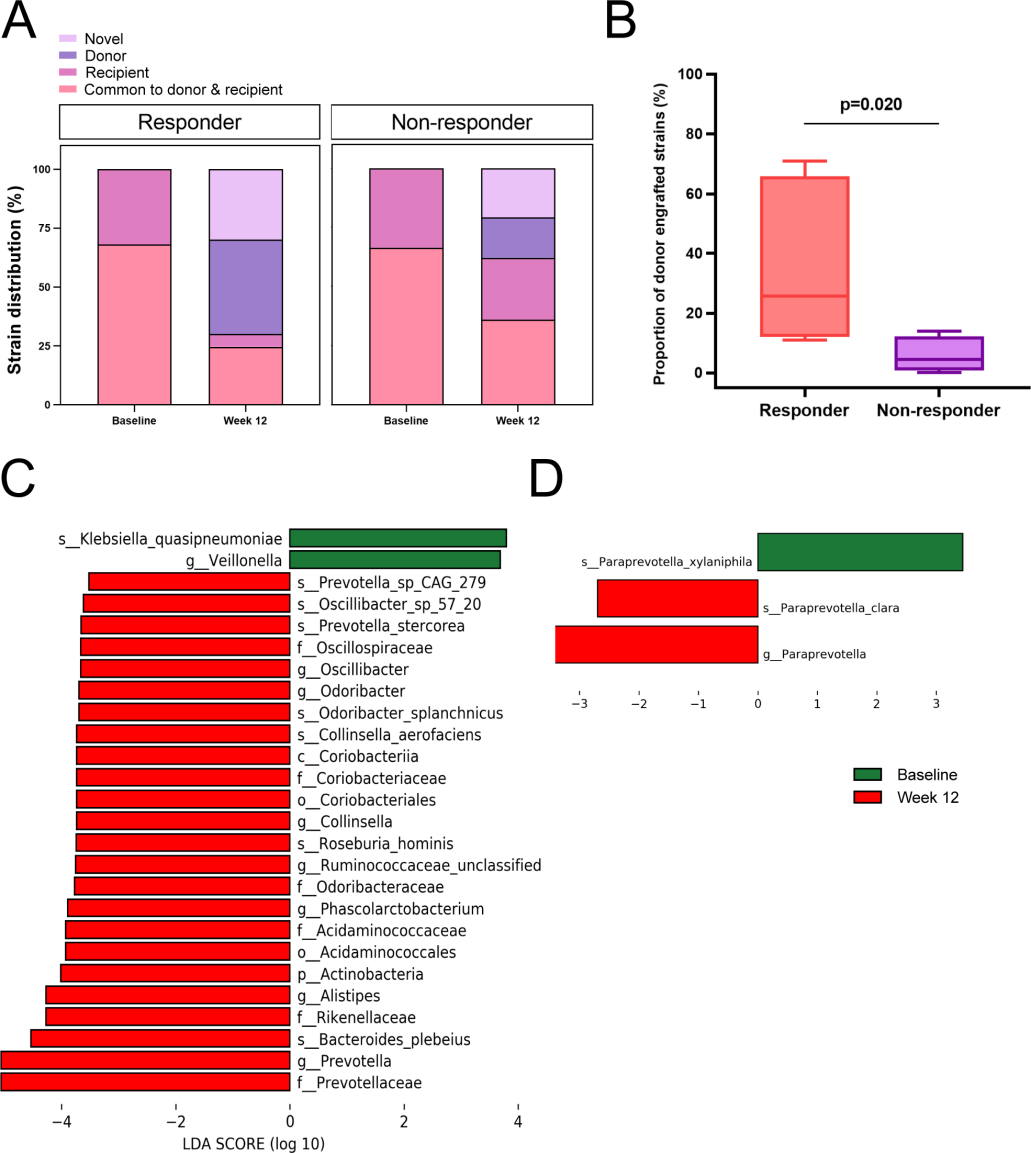
Strain composition in responders and non-responders at baseline and post-FMT. (**A**) Strain distribution across groups. “Novel” refers to newly observed strains not matched to recipient baseline or donor strains. “Donor” and “recipient” denote strains unique to donors or recipients, respectively, while “common to donor and recipient” refers to shared strains. (**B**) Proportion of donor-engrafted strains. Data were analyzed by an independent *t*-test. (C and D) Multilevel species differences between pre- and post-FMT samples in responders and non-responders were identified using linear discriminant analysis effect size (LEfSe). Taxa were analyzed using a non-parametric Kruskal-Wallis test (*P* < 0.05, linear discriminant analysis [LDA] score >2).

To investigate whether clinical response correlates with the origins of microbial strains, post-transplantation strains in recipients were categorized into four groups. The distribution of strains in responders and non-responders was compared before and after FMT (Fig. 4A). To further specify the taxonomic composition of different strain categories in responders and non-responders, we summarized the top taxa in each category in Tables S4 and S5. Individual variability in the distribution of strains at baseline and week 12 is detailed in Supplementary Figures (Fig. S3). By week 12, 37.8% of strains in the responder group originated from donors, compared to only 15.2% in the non-responder group. The higher proportion of donor-derived strains in responders highlights the potential role of strain origin in influencing clinical efficacy (Fig. 4B; *P* = 0.020).

LEfSe analysis was performed to identify specific taxa significantly altered by FMT, particularly those contributing to weight loss in responders. Key taxa enriched post-FMT in responders included *Actinobacteria*, *Odoribacteraceae*, *Prevotellaceae*, *Oscillospiraceae*, *Rikenellaceae*, and *Acidaminococcales* (Fig. 4C). Conversely, *Paraprevotella* was significantly enriched in non-responders following FMT (Fig. 4D), providing insights into distinct microbial shifts associated with differing clinical outcomes.

### Broad microbial dynamics and key taxa associated with clinical response

**Fig 5:**
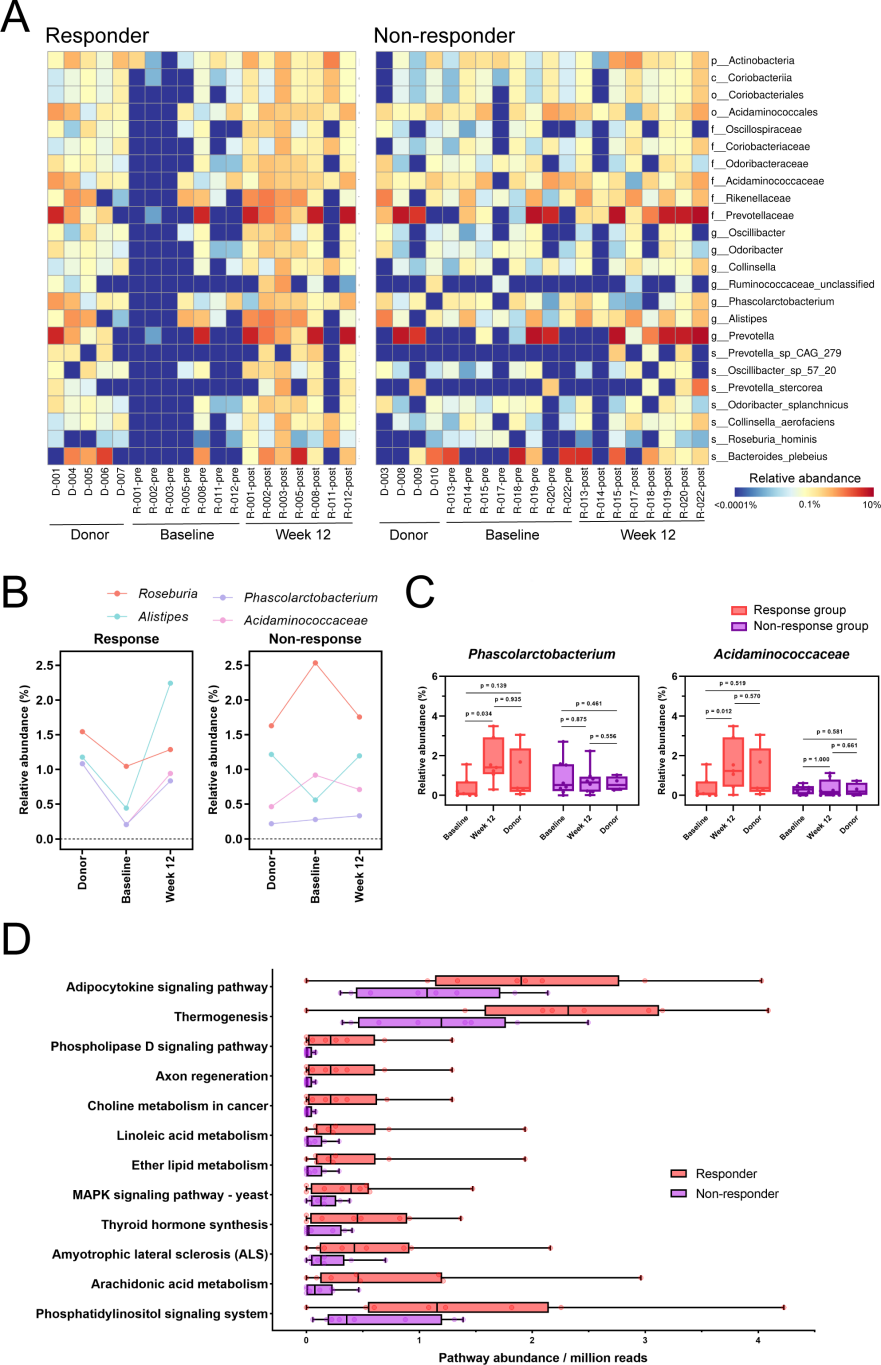
Shifts in gut microbiome composition toward matched donor following FMT. (**A**) Heatmap depicting the relative abundance of key taxa. Data were log_10_-transformed for visualization. (**B**) The mean relative abundance of *Roseburia*, *Alistipes*, *Phascolarctobacterium*, and *Acidaminococcaceae* in donors and recipients at baseline and in recipients post-FMT. (**C**) Relative abundance of *Phascolarctobacterium* and *Acidaminococcaceae* in donors and recipients (responders and non-responders) at baseline and post-FMT. The *P* values were obtained from LEfSe (using non-parametric Kruskal-Wallis tests). (**D**) Enriched bacterial metabolic pathways in the gut microbiome of responders post-FMT, showing only pathways with statistically significant differences (*P* < 0.05). Statistical analyses were performed using paired and unpaired *t*-tests for normally distributed data, and Mann-Whitney *U* or Wilcoxon signed-rank tests for non-normally distributed data. FDR-adjusted *P* values are denoted.

To explore global microbial dynamics and identify key taxa potentially contributing to weight loss outcomes, heatmaps were generated to visualize the relative abundances of multiple bacterial taxa in donors, responders, and non-responders at baseline and 12 weeks (Fig. 5A). These findings revealed distinct microbial shifts in responders that aligned more closely with donor microbiota post-FMT. This pattern was absent in the non-responders, suggesting that specific taxa may play a pivotal role in the observed weight loss. Additionally, heatmaps for each donor-recipient pairing were provided to further illustrate individual microbial dynamics (Fig. S4).

We next determined whether successful weight loss in responders was attributable to the colonization of specific donor taxa. Specifically, we focused on four key taxa: *Roseburia*, *Alistipes*, *Phascolarctobacterium*, and *Acidaminococcaceae*, chosen for their patterns and potential relevance to gut microbiota-mediated metabolic functions. These taxa were enriched in donors and showed increased mean abundance in responders post-FMT (Fig. 5B). Among them, *Phascolarctobacterium* (*P* = 0.034) and *Acidaminococcaceae* (*P* = 0.012) demonstrated significant increases in responders post-FMT (Fig. 5C), indicating successful engraftment and their potential functional contributions to weight loss. Notably, the abundance of these taxa did not differ significantly between donors and recipients at baseline or at 12 weeks (*P* > 0.05 for both), underscoring that the therapeutic effect in responders was primarily driven by the successful establishment and expansion of these taxa post-FMT, rather than the donor’s baseline abundance.

In contrast, non-responders showed no significant changes in the abundance of these four taxa pre- and post-FMT, highlighting the lack of microbial shifts or engraftment in this group. Additionally, a comparison of the relative abundance of these taxa in donors between the responder and non-responder groups revealed no significant difference (Fig. S5). These findings emphasize the critical role of successful engraftment, rather than donor abundance, in mediating the observed clinical benefits in responders.

### Distinct metabolic pathway alterations associated with FMT response

FMT led to distinct metabolomic alterations between responders and non-responders. To explore these differences, we analyzed the metabolic pathways of gut microbiota. No significant differences were observed between the two groups at baseline (Fig. S6). By week 12, however, 12 pathways were significantly enriched in responders (Fig. 5D). These pathways included thermogenesis, axon regeneration, choline metabolism, linoleic acid metabolism, ether lipid metabolism, thyroid hormone synthesis, and arachidonic acid metabolism. The fecal metagenomes of responders exhibited an enhanced capacity for these metabolic functions, suggesting sustained functional alterations in microbial communities induced by FMT. Importantly, the enrichment of these specific pathways, such as adipocytokine signaling pathway, thyroid hormone synthesis, and thermogenesis, likely contributes to the observed weight loss in responders. These findings highlight distinct functional microbial shifts in responders and underscore their potential as metabolic targets for enhancing FMT efficacy.

To further explore the relationship between microbial taxa and functional changes associated with weight loss, we analyzed the associations between four strain types (donor-derived, common, novel, and recipient only) and the metabolic pathways enriched in responders following FMT. The results showed that these pathways were primarily associated with donor-derived, common, and novel genera, suggesting that functional shifts after FMT are driven by contributions from multiple microbial sources (Fig. S7).

## DISCUSSION

This study builds upon existing evidence supporting the therapeutic potential of FMT for obesity management through the modulation of gut microbiota. Previous research has reported inconsistent outcomes regarding FMT’s impact on weight loss. For instance, one study found no significant weight changes in obese patients following FMT administered via frozen capsules (15), while another reported modest weight reduction after advanced antibiotic therapy and FMT delivered via esophagogastroduodenoscopy (13). Our findings demonstrate that FMT through a nasojejunal tube is an effective intervention for achieving significant weight loss in overweight and obese individuals, with donor-derived microbial engraftment playing a critical role in these outcomes.

To better understand the variability in FMT efficacy, we first noted that recipients with the same donor tended to have similar weight-loss outcomes, suggesting that donor characteristics may play a role in therapeutic variability. We compared microbial diversity and functional pathways between donors linked to responders and non-responders. Although donors corresponding to non-responders tended to have lower alpha diversity compared with those linked to responders, these differences did not reach statistical significance. Additionally, no significant differences were observed in functional pathways between these donor groups. We further explored whether other donor characteristics, including age, BMI, and metabolic parameters, correlated with recipient response status (Table S2), but none of these factors demonstrated statistically significant associations. Therefore, while these observations indicate that donor microbiota composition alone might not fully explain recipient weight loss outcomes, our conclusions remain cautious due to the limited sample size, which restricts statistical power and could mask subtle but biologically meaningful effects. Larger studies are warranted to clarify the precise contribution of donor-related factors to recipient outcomes.

Unlike studies that reported a generalized increase in microbial diversity post-FMT (11), we observed a trend toward higher alpha and beta diversity post-FMT among responders, though the differences were not statistically significant after multiple testing corrections. This trend likely reflects biologically meaningful changes that could not be captured due to the study’s sample size. Increased alpha diversity in responders aligns with the hypothesis that a more diverse microbiota enhances metabolic stability and functional capacity, while shifts in beta diversity indicate successful integration of donor-derived strains into the recipient gut ecosystem.

A key finding of our study is the significant engraftment of specific donor-derived taxa, such as *Phascolarctobacterium* and *Acidaminococcaceae*, in responders post-FMT. These taxa, known for their metabolic roles, demonstrated significant increases despite no notable differences in baseline abundance between donors and recipients. This highlights the importance of engraftment efficiency rather than donor composition alone in driving therapeutic outcomes post-FMT. Our findings are consistent with studies in other contexts, such as *Clostridioides difficile* infection, where successful engraftment of nearly 40% of donor taxa was associated with clinical remission (16). In contrast, non-responders exhibited an absence of microbial shifts, emphasizing the critical role of the recipient gut environment in enabling colonization and functional activation of donor-derived microbes. Moreover, beta diversity analysis revealed limited overall microbial convergence between donors and paired recipients post-FMT. This observation reinforces the idea that therapeutic effects may rely more on the functional activity of engrafted taxa than on broad compositional similarities.

The recipient’s gut environment plays a pivotal role in determining FMT outcomes. For instance, available niches in the recipient’s gut and favorable interactions with the existing microbial community might facilitate colonization (17), providing a conducive environment for the colonization and proliferation of specific taxa. Conversely, non-responders may have a more resistant baseline microbiome or an inhospitable gut environment, limiting successful microbial engraftment. Interventions such as dietary preconditioning or prebiotic supplementation could enhance engraftment in such cases. Additionally, factors such as recipient immune tolerance (18) and host-microbial cross-talk (19) may influence the engraftment process. Future studies are needed to investigate specific microbial traits, such as adhesion mechanisms or metabolic adaptability, that contribute to successful colonization.

Mechanistically, the functional roles of engrafted taxa provide insights into the observed weight loss. *Phascolarctobacterium* produces short-chain fatty acids (SCFAs) like propionate, which are associated with improved energy metabolism, appetite regulation, reduced inflammation (20), and improved insulin sensitivity (21). Similarly, *Acidaminococcaceae* is involved in amino acid metabolism and SCFA production, which collectively improve gut barrier integrity, reduce systemic inflammation, and regulate lipid and glucose metabolism (22–24). Functional metagenomic analysis further revealed distinct enrichment of metabolic pathways related to thermogenesis, lipid metabolism, thyroid hormone synthesis, and arachidonic acid metabolism in responders. Thermogenesis and lipid metabolism pathways are particularly relevant, as they influence energy expenditure and fat utilization. Similarly, thyroid hormones are key regulators of metabolic rate, while arachidonic acid metabolism is involved in the production of bioactive lipids that modulate inflammation and lipid metabolism. Interestingly, our correlation analysis revealed that the metabolic pathways enriched in responders post-FMT were significantly associated not only with donor-derived taxa but also with taxa common to both donors and recipients, as well as novel taxa that emerged following FMT. These findings suggest that functional shifts in the gut microbiome are not solely attributable to the engraftment of donor-derived microbes but rather involve complex interactions among donor-derived microbes and resident recipient taxa. The significant correlation with common taxa further indicates the collective contributions of multiple microbial sources acting in concert to reprogram host metabolic functions. This observation aligns with previous reports indicating that microbial community function often emerges from synergistic interactions among coexisting strains, rather than the dominance of a single taxonomic group (16, 25). Moreover, the correlation with novel taxa highlights the possibility that FMT creates a reshaped microbial ecosystem that enables the emergence of new functional niches and reshapes the microbial ecosystem, contributing substantially to the functional reprogramming associated with therapeutic responses.

These results have significant clinical implications. While donor diversity alone is insufficient to predict FMT efficacy, selecting donors enriched with specific functional taxa, such as *Phascolarctobacterium* and *Acidaminococcaceae*, may enhance therapeutic outcomes rather than broad compositional characteristics. Moreover, the lack of microbial shifts in non-responders highlights the necessity of developing strategies to optimize the recipient gut environment, such as pre-treatment interventions to improve niche compatibility or co-administration of adjunctive therapies to support microbial engraftment. Investigating host factors, including genetics, baseline microbiota composition, and immune response, could further elucidate the variability in FMT outcomes.

This study has several limitations. The lack of a placebo group limits the ability to attribute weight loss to FMT, and the relatively small sample size reduces the statistical power to detect subtle but meaningful microbial changes. Additionally, while microbial composition changes were observed, the causal role of specific donor-derived taxa remains speculative. Further studies should employ larger, controlled cohorts to validate these findings and clarify the pathways through which engrafted taxa influence weight loss.

In conclusion, our findings highlight the critical role of donor-derived microbial engraftment in driving FMT efficacy for weight loss. This study underscores the importance of specific taxa, such as *Phascolarctobacterium* and *Acidaminococcaceae*, in promoting weight loss through functional reprogramming of the gut microbiota. These results provide a strong foundation for developing personalized microbiota-targeted therapies in obesity management, emphasizing the need for refined donor selection and recipient preconditioning to optimize therapeutic outcomes.

## MATERIALS AND METHODS

### Study design

This single-arm, multi-center intervention trial investigated the efficacy of fecal microbiota transplantation in overweight and obese individuals. A total of 25 participants received fresh fecal flora fluids from 10 healthy donors. Each participant underwent three FMT sessions and administered biweekly, and adverse events were monitored throughout the study. Stool samples were collected at baseline and 8 weeks after the final sessions (Fig. 1A).

### Participants and FMT intervention

Participants aged 18–65 years with a BMI ≥24 kg/m^2^ and stable body weight (<10% fluctuation over the preceding 6 months) were included. Exclusion criteria included uncontrolled hypertension (systolic blood pressure ≥180 mmHg or diastolic blood pressure ≥110 mmHg), hypertriglyceridemia, recent insulin use, acute infections, immunosuppression (due to organ transplantation, HIV, cancer chemotherapy, or immunosuppressive agents usage), malignancy, pregnancy, poorly controlled type 2 diabetes mellitus, non-alcoholic steatohepatitis, or metabolic syndrome. Written informed consent was obtained from all participants and donors.

Throughout the study, participants were instructed to adhere to their regular dietary and exercise routines. FMT was performed biweekly for three sessions using 200 mL of fresh fecal microbiota solution delivered via a nasojejunal tube into the duodenum. Participants were advised to remain upright at a 45° angle for at least 4 hours post-infusion to minimize aspiration risk (15, 26). Detailed participant and donor profiles are provided in Table S1.

### Donor selection and preparation of FMT solution

Donors aged ≥14 years with a BMI between 18.5 and 23.9 kg/m^2^ underwent comprehensive screening, including medical history, physical examination, and laboratory testing for infectious and metabolic diseases. Recent antibiotic use or gastrointestinal infections within 3 months were exclusion criteria (15). Fresh stool samples were diluted in sterile physiological saline, homogenized, filtered, and centrifuged to isolate bacterial pellets. These pellets were resuspended in saline containing 12.5% glycerol as a cryoprotectant (27, 28). All steps were performed under strict anaerobic conditions to preserve anaerobes. The final product was stored at −80°C until use (11).

### Outcome measures

Vital signs, including body temperature, blood pressure, respiration, and heart rate, were monitored for 24 hours post-FMT. Weekly follow-up calls were conducted to record adverse events. Clinical assessments included body weight, BMI, body fat percentage, and waist circumference at baseline and 12 weeks. Participants also completed a 3-day dietary record at 12 weeks. Responders were defined as participants achieving ≥5% wt loss, while non-responders had <5% wt loss. Secondary outcomes included microbial composition analysis via metagenomic sequencing to identify differences among donors, responders, and non-responders.

### DNA extraction and library preparation

Stool samples were collected aseptically and transported on ice to the laboratory within 2 hours of collection. DNA extraction was performed using Qiagen PowerBead tubes, Qiagen C1 buffer, and EZ1 DNA Tissue Kits on the Qiagen EZ1 Advanced XL platform. Prior to extraction, stool samples were homogenized, and bacterial cells were lysed with lysozyme and lysostaphin. Sequencing libraries were prepared using the NexteraXT DNA Library Preparation Kit (Illumina) and sequenced on an Illumina NovaSeq 6000 platform using a paired-end protocol (2 × 150 bp). The average sequencing depth was approximately 8 million reads per sample for metagenomic analysis.

### Strain-level analysis

Strain-level comparisons were conducted using StrainPhlAn, leveraging single nucleotide polymorphism (SNP)-based analysis of core genes. Metagenomic reads were mapped to the MetaPhlAn3 reference database using Bowtie2. To evaluate strain transplantation, strains were classified into four categories based on their presence in donors and recipients. “Novel” strains were defined as newly observed strains not matching either the recipient’s baseline or donor strains. “Recipient” strains were detected in the recipient at baseline but absent in the donor. “Donor” strains were detected only in the donor sample, were absent in the baseline recipient sample, and significantly increased in abundance post-FMT. “Common to donor and recipient” strains were shared between donors and recipients. Engraftment was defined as the detection of either “donor” or “common to donor and recipient” strains in the recipient sample post-FMT, with a statistically significant increase in abundance compared to baseline. LEfSe analysis was performed to identify taxonomic groups with differential relative abundance across samples, and results were visualized using heatmaps.

### Metagenomic and taxonomic analyses

Metagenomic reads underwent quality control using FastQC and Trimmomatic, followed by human read removal via KneadData. Taxonomic classification was performed using MetaPhlAn3, generating relative abundance tables directly from metagenomic reads without assembly. Strain-level classifications were based on SNP analysis. Data visualization and relative abundance calculations were performed using RStudio v1.3.1056, with tidyverse v1.3.0, vegan v2.5.7, and phyloseq v1.34.0 packages. Differences in microbial composition were evaluated using permutational multivariate analysis of variance (PERMANOVA, adonis2 in vegan), and results were visualized using principal coordinate analysis (PCoA) with ggplot2 and ggrepel.

### Assembly-based genomic analysis

Metagenomic reads were assembled into contigs using metaSPAdes (v3.14.1). Genome-wide average nucleotide identity was calculated with FastANI, and pangenome analyses were performed using Roary. Metagenome-assembled genomes were binned with MaxBin2 and MetaBAT2, followed by dereplication with DAS Tool. Taxonomic classification was conducted using GTDB-Tk. Reads were competitively mapped to dereplicated genomes with Bowtie2, and strain presence was confirmed using a coverage threshold of ≥50%.

### Alpha and beta diversity

Alpha diversity indices, including Shannon index and richness, were calculated using phyloseq in R. Beta diversity was assessed with Bray-Curtis dissimilarity and visualized using PCoA. Differences in microbial composition were evaluated using PERMANOVA, with *P*-values adjusted using the Benjamini-Hochberg method.

### Functional and metabolic pathway analysis

Metabolic function profiles were estimated using HUMAnN2 and the Kyoto Encyclopedia of Genes and Genomes (KEGG) database. Functional pathways were quantified by mapping metagenomic reads to the KEGG reference database, and results were compared across groups to identify functional changes associated with FMT outcomes.

### Statistical analyses

An intention-to-treat analysis was conducted with multiple imputations for missing data. Normally distributed data were analyzed using paired and unpaired *t*-tests, while non-normally distributed data were assessed using Mann-Whitney *U* and Wilcoxon signed-rank tests. Correlation analyses were performed using Pearson correlation for normally distributed data and Spearman correlation for non-normally distributed data. To control for multiple comparisons, *P* values were adjusted using the Benjamini-Hochberg method, with *q* < 0.05 considered statistically significant. Statistical analyses were performed using SPSS (v25, IBM Corp) and R.

## Data Availability

All metagenomic sequences have been deposited in the European Bioinformatics Institute-Sequence Read Archive database, under accession number PRJNA1243589.

## SUPPLEMENTAL MATERIAL

Supplemental material aem.00120-25-s0001.pdf Tables S1 to S5; Fig. S1 to S7.

ASM does not own the copyrights to Supplemental Material that may be linked to, or accessed through, an article. The authors have granted ASM a non-exclusive, world-wide license to publish the Supplemental Material files. Please contact the corresponding author directly for reuse.
