# Supplementary material for: Donor-derived microbial engraftment and gut microbiota shifts associated with weight loss following fecal microbiota transplantation
Source: Appl Environ Microbiol. 2025 Jun 4;91(7):e00120-25. doi: 10.1128/aem.00120-25 (PMC12285249; doi:10.1128/aem.00120-25)
Supplement: Supplemental material — Tables S1 to S5; Fig. S1 to S7. [file aem.00120-25-s0001.pdf]

Supplementary Table S1 Characteristics of donors at baseline

	#1	#2	#3	#4	#5	#6	#7	#8	#9	#10
**Age**	25	17	25	20	15	21	23	24	19	20
**Sex**[1]	F	F	M	F	F	M	F	F	M	F
**Body weight (kg)**	45	50	57	55	52	56	55	51	57	53
**Initial BMI (kg/m^2)**	18.49	19.29	19.72	19.72	21.10	19.38	22.60	18.51	19.05	18.34
**Waist (cm)**	65	67	75	64	70	71	62	60	63	60
**Waist-hip ratio**	0.76	0.78	0.81	0.75	0.78	0.83	0.72	0.76	0.74	0.66
**SBP (mmHg)**	115	128	120	109	126	120	126	118	111	110
**DBP (mmHg)**	72	80	71	72	87	77	81	71	61	66
**Fasting blood glucose (mmol/L)**	5.29	4.37	5.02	4.51	4.26	4.12	4.85	4.62	3.95	5.14
**Fasting insulin (mIU/L)**	3.90	9.60	6.62	6.30	8.60	12.21	10.80	9.66	4.85	6.21
**AST (IU/L)**	18.56	21.52	19.14	13.89	22.91	20.12	16.52	27.51	17.52	22.85
**ALT (IU/L)**	9.04	15.45	16.37	11.15	16.49	14.00	14.88	24.11	16.60	19.84
**Scr (umol/L)**	61.96	50.01	81.39	57.63	51.84	80.00	54.66	57.05	76.70	73.11
**BUN (mmol/L)**	3.15	2.67	4.03	3.37	3.16	3.46	3.25	3.35	4.75	4.72

**Uric acid (umol/L)**	240.01	221.62	317.69	241.03	295.15	335.00	339.29	259.66	325.95	239.61
**TG (mmol/L)**	0.48	1.49	0.70	0.83	1.68	0.84	0.39	0.53	0.72	0.45
**TC (mmol/L)**	4.56	3.40	4.58	4.64	4.79	3.72	3.81	4.05	3.26	3.72
**HDL-C (mmol/L)**	1.63	1.30	1.30	1.24	1.35	1.37	1.34	1.41	0.90	1.44
**LDL-C (mmol/L)**	2.50	1.92	2.81	3.11	3.17	2.41	2.18	2.41	2.01	2.30

Abbreviations BMI, body mass index; BFR, body fat rate; SBP, systolic blood pressure; DBP, diastolic blood pressure; TG, triglyceride; AST, aspartate aminotransferase; ALT, alanine aminotransferase; Scr, serum creatinine; BUN, blood urea nitrogen; TC, total cholesterol; HDL-C, high density lipoprotein cholesterol; LDL-C, low density lipoprotein cholesterol.

[1]F, female; M, male.

Supplementary Table S2 Baseline characteristics of donors associated with responders and non-responders

**Characteristic**	**Response (n = 5)**	**Non-response (n = 5)**	***P* value**
Age	20.40 (4.56)	21.40 (2.07)	0.964
Female, No. (%)	4 (80%)	3 (60%)	0.841
Body weight (kg)	51.80 (4.66)	54.40 (2.41)	0.841
Initial BMI (kg/m^2)	19.66 (0.95)	19.58 (1.74)	0.529
Waist (cm)	68.20 (4.44)	63.20 (4.55)	0.841
Waist-hip ratio	0.78 (0.02)	0.74 (0.06)	0.841
SBP (mmHg)	119.60 (7.83)	117.00 (6.63)	0.841
DBP (mmHg)	76.40 (6.95)	71.20 (8.07)	0.529
Fasting blood glucose (mmol/L)	4.69 (0.44)	4.54 (0.50)	0.964
Fasting insulin (mIU/L)	7.00 (2.21)	8.75 (3.11)	0.841
AST (IU/L)	19.20 (3.46)	20.90 (4.44)	0.529
ALT (IU/L)	13.70 (3.40)	17.89 (4.13)	0.529
Scr (umol/L)	60.57 (12.57)	68.30 (11.65)	0.841
BUN (mmol/L)	3.28 (0.49)	3.91 (0.76)	0.529
Uric acid (umol/L)	263.10 (41.07)	299.90 (46.68)	0.841
TG (mmol/L)	1.04 (0.52)	0.59 (0.19)	0.843

TC (mmol/L)	4.39 (0.56)	3.71 (0.29)	0.529
HDL-C (mmol/L)	1.36 (0.15)	1.29 (0.22)	0.841
LDL-C (mmol/L)	2.70 (0.51)	2.26 (0.17)	0.529

Abbreviations BMI, body mass index; BFR, body fat rate; SBP, systolic blood pressure; DBP, diastolic blood pressure; TG, triglyceride; AST, aspartate aminotransferase; ALT, alanine aminotransferase; Scr, serum creatinine; BUN, blood urea nitrogen; TC, total cholesterol; HDL-C, high density lipoprotein cholesterol; LDL-C, low density lipoprotein cholesterol.

Donors were grouped according to the response status of their corresponding recipients. Data are presented as mean (SD) or as the number of subjects (%). *P* values were assessed by unpaired t-test for data with a normal distribution or Mann-Whitney U test for data with a non-normal distribution between the two groups.

Supplementary Table S3 Characteristics of fecal microbiomes in donors at baseline

Donor ID	Shannon Index	Richness Index	Dominant Taxa (Top 5 Genera)	Relative Abundance (%)	Short-Chain Fatty Acid (SCFA) Producers(%)
D1	2.5	62	*Prevotella, Tobamovirus, Parabacteroides, Phascolarctobacterium, Coprococcus*	53, 8, 7, 6, 5	27.9
D2	2.7	46	*Bacteroides, Lachnospiraceae unclassified, Ruminococcus, Roseburia, Faecalibacterium*	30, 20, 16, 10, 9	36.7
D3	2.8	45	*Bacteroides, Alistipes, Escherichia, Faecalibacterium, Parabacteroides*	77, 6, 4, 3, 2	55.4
D4	2.7	100	*Escherichia, Bacteroides, Alistipes, Faecalibacterium, Parabacteroides*	38, 24, 5, 5, 4	20.0
D5	2.9	90	*Bacteroides, Faecalibacterium, Lachnospiraceae unclassified, Escherichia, Firmicutes unclassified*	42, 13, 9, 8, 5	25.5
D6	2.5	71	*Bacteroides, Escherichia,*	60, 12, 6, 4, 4	29.7

			Faecalibacterium, Lachnospiraceae unclassified, Ruminococcus		
D7	2.7	56	*Bacteroides, Faecalibacterium, Lachnospiraceae unclassified, Roseburia, Parabacteroides*	52, 12, 11, 6, 4	35.9
D8	1.3	62	*Prevotella, Bacteroides, Faecalibacterium, Lachnospira, Parabacteroides*	74, 10, 3, 3, 1	43.3
D9	1.0	38	*Prevotella, Bacteroides, Lachnospiraceae unclassified, Phascolarctobacterium, Eubacterium*	85, 5, 3, 1, 1	20.1
D10	2.9	84	*Bacteroides, Escherichia, Faecalibacterium, Parabacteroides, Klebsiella*	34, 28, 6, 6, 4	19.8

Supplementary Table S4 Genus-level taxa classified by dominant strain category in responders after FMT

Taxon	Responder (% in subjects)[1]	Strain Category[2]	Function
Collinsella	71%	Donor	Butyrate production, bile acid metabolism
Alistipes	57%	Donor	SCFA production
Dorea	57%	Donor	Anti-inflammatory, acetate and proprionate production
Odoribacter	57%	Donor	SCFA production
Oscillibacter	57%	Donor	Maintaining mucosal homeostasis, anti-inflammatory
Megamonas	57%	Donor	Glycometabolism and muscle growth
Anaerostipes	43%	Donor	Acetate and butyrate production
Ruminococcus	43%	Donor	Polysaccharide breakdown
Ruminococcaceae unclassified	43%	Donor	Fiber degradation

Bacteroides	86%	Common	Polysaccharide degradation
Blautia	86%	Common	Acetate and butyrate production
Faecalibacterium	71%	Common	SCFA production
Eubacterium	71%	Common	Butyrate and bile acid metabolism
Roseburia	71%	Common	SCFA production
Fusicatenibacter	71%	Common	Anti-inflammatory, SCFA production
Parabacteroides	57%	Common	SCFA production
Phascolarctobacterium	57%	Common	SCFA production
Flavonifractor	57%	Common	Flavonoid metabolism
Agathobaculum	57%	Common	SCFA production
Lachnospiraceae unclassified	57%	Common	Butyrate production

Bifidobacterium	57%	Common	Lactate and acetate production
Escherichia	43%	Common	Mixed acid fermentation
Streptococcus	43%	Common	Lactate production
Intestinimonas	57%	Novel	Butyrate production
Ruthenibacterium	43%	Novel	SCFA production
Anaerotruncus	43%	Novel	SCFA production
Lachnoclostridium	43%	Novel	Butyrate production
Fusobacterium	43%	Novel	Amino acid fermentation
Lawsonibacter	43%	Novel	SCFA production
Sutterella	43%	Novel	Glutathione metabolism
Firmicutes unclassified	43%	Novel	SCFA production
Klebsiella	43%	Recipient	Ethanol production
Enterococcus	14%	Recipient	Lactate production

Acinetobacter	14%	Recipient	Organic compound degradation

Abbreviations SCFA, Short-chain fatty acid

[1]% in subjects indicates the proportion of donor-recipient pairs in which the genus was assigned to the corresponding dominant category.

[2]Representative genus was assigned to a dominant strain type (donor-derived, common, novel, or recipient-only) based on the category in which it most frequently appeared across donor-recipient pairs in the responder group. “Novel” refers to newly observed strains not matched to recipient baseline or donor strains. “Donor” and “Recipient” denote strains unique to donors or recipients, respectively, while “Common” refers to shared strains.

Supplementary Table S5 Genus-level taxa classified by dominant strain category in non-responders after FMT

Taxon	Non-responder (% in subjects)[1]	Strain Category[2]	Function
Paraprevotella	50%	Donor	Polysaccharide degradation
Coprobacter	38%	Donor	Acetate and proprionate production
Clostridium	38%	Donor	Butyrate production
Barnesiella	25%	Donor	Anti-inflammatory
Akkermansia	13%	Donor	Maintenance of intestinal barrier
Bacteroides	100%	Common	Polysaccharide degradation
Parabacteroides	100%	Common	SCFA production
Alistipes	88%	Common	SCFA production
Dorea	88%	Common	Anti-inflammatory, acetate and proprionate production
Faecalibacterium	88%	Common	SCFA production

Eubacterium	88%	Common	Butyrate and bile acid metabolism
Roseburia	88%	Common	SCFA production
Blautia	75%	Common	Acetate and butyrate production
Escherichia	75%	Common	Mixed acid fermentation
Fusicatenibacter	63%	Common	Anti-inflammatory, SCFA production
Phascolarctobacterium	63%	Common	SCFA production
Odoribacter	50%	Common	SCFA production
Flavonifractor	50%	Common	Flavonoid metabolism
Coprococcus	50%	Common	SCFA production
Collinsella	38%	Common	Butyrate production, bile acid metabolism
Oscillibacter	38%	Common	Maintaining mucosal homeostasis, anti-inflammatory
Agathobaculum	38%	Common	SCFA production
Ruthenibacterium	38%	Novel	SCFA production

Lachnospira	38%	Novel	SCFA production
Lactobacillus	38%	Novel	SCFA production
Holdemania	38%	Novel	SCFA production
Actinomyces	38%	Novel	Carbohydrate fermentation
Anaerostipes	50%	Recipient	Acetate and butyrate production
Streptococcus	50%	Recipient	Lactate production
Lachnospiraceae unclassified	38%	Recipient	Butyrate production
Acidaminococcus	38%	Recipient	Amino acid fermentation
Prevotella	38%	Recipient	Polysaccharide degradation
Butyricimonas	38%	Recipient	Butyrate production

Abbreviations SCFA, Short-chain fatty acid

[1]% in subjects indicates the proportion of donor-recipient pairs in which the genus was assigned to the corresponding dominant category.

[2]Representative genus was assigned to a dominant strain type (donor-derived, common, novel, or recipient-only) based on the category in which it most frequently appeared across donor-recipient pairs in the non-responder group. “Novel” refers to newly observed strains not matched to recipient baseline or donor strains. “Donor” and “Recipient” denote strains unique to donors or recipients, respectively, while “Common” refers to shared strains.

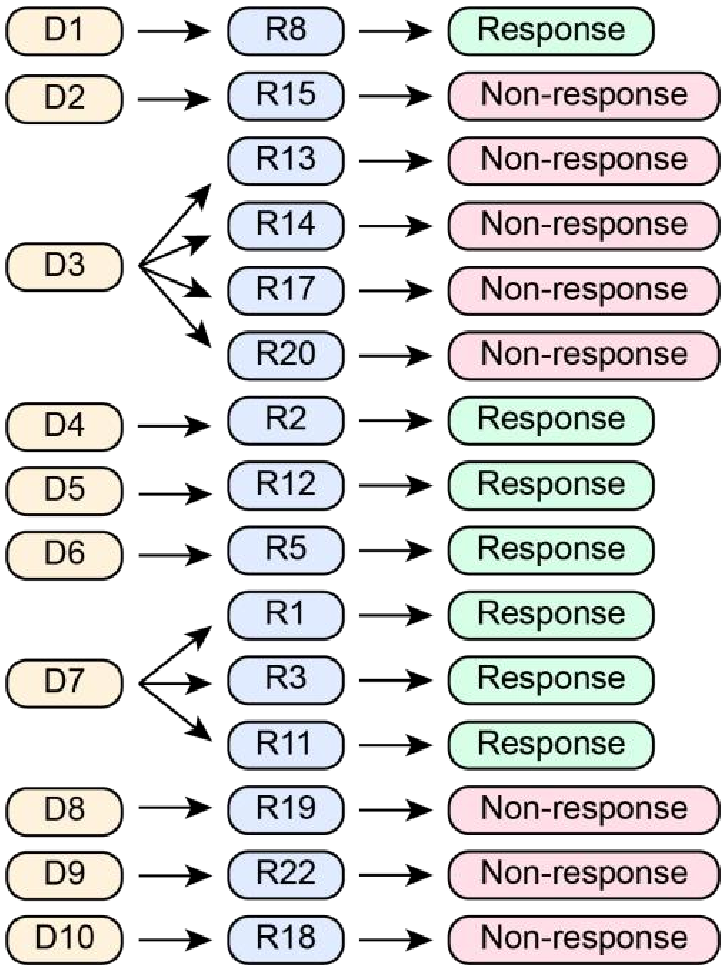

Fig. S1 Pairing diagram illustrating donor-recipient matching.

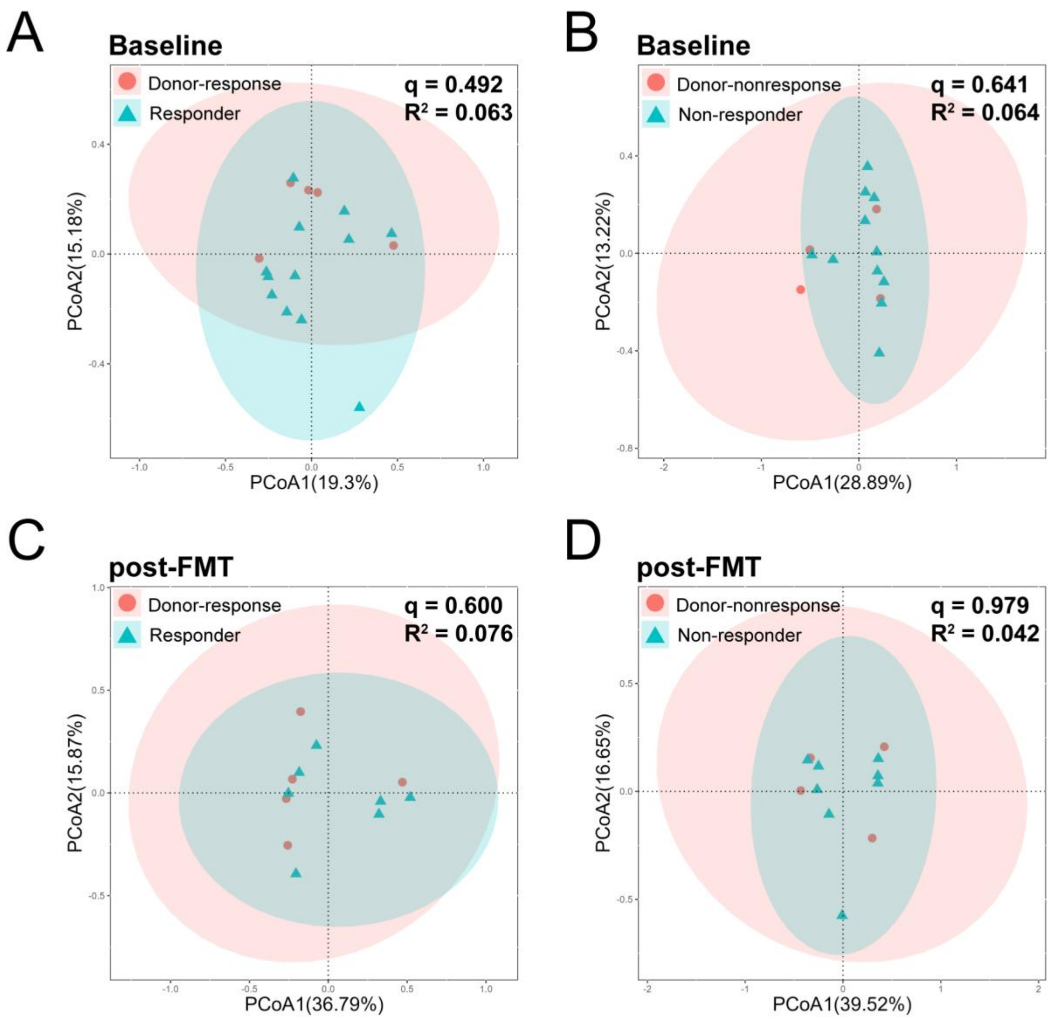

Fig. S2 Beta-diversity analysis of donors and their matching recipients. Comparisons were conducted between donors and recipients at baseline (**A, B**) and post-FMT (**C, D**). Principal Coordinates Analysis (PCoA) was performed using Bray-Curtis dissimilarity data at the species level. *P* values were adjusted using the Benjamini-Hochberg false discovery rate (FDR) method ($^{*}q < 0.05$). The low R^2-value indicates weak overall grouping.

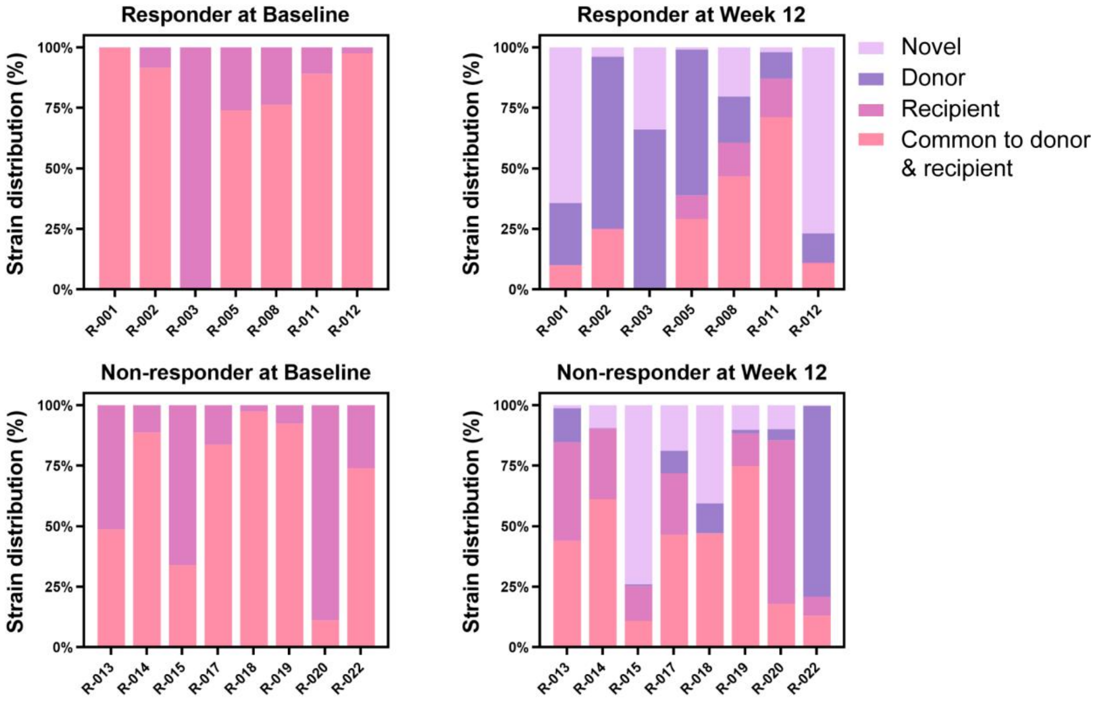

Fig. S3 Strain composition in responders and non-responders at baseline and 12 weeks. "Novel" refers to newly observed strains not matched to recipient baseline or donor strains. "Donor" and "Recipient" denote strains unique to donors or recipients, respectively, while "Common to donor & recipient" refers to shared strains.

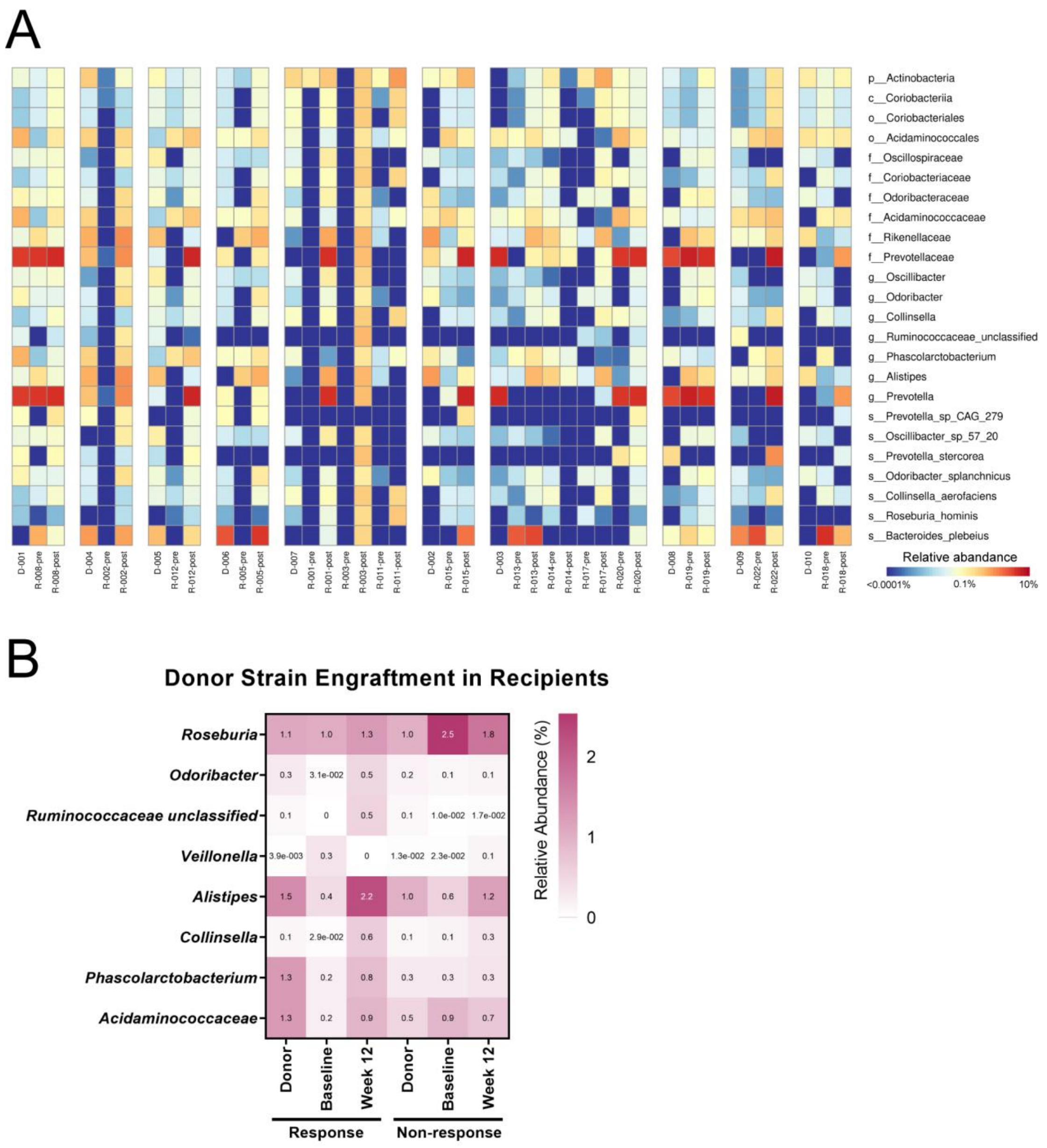

Fig. S4 Shifts in gut microbiome composition towards matched donor strains in each recipient. (**A**) Heatmap depicting the relative abundance of taxa for each donor-recipient pair. Data were $\log_{10}$-transformed for visualization. (**B**) Relative abundance of eight taxa enriched in responders post-FMT.

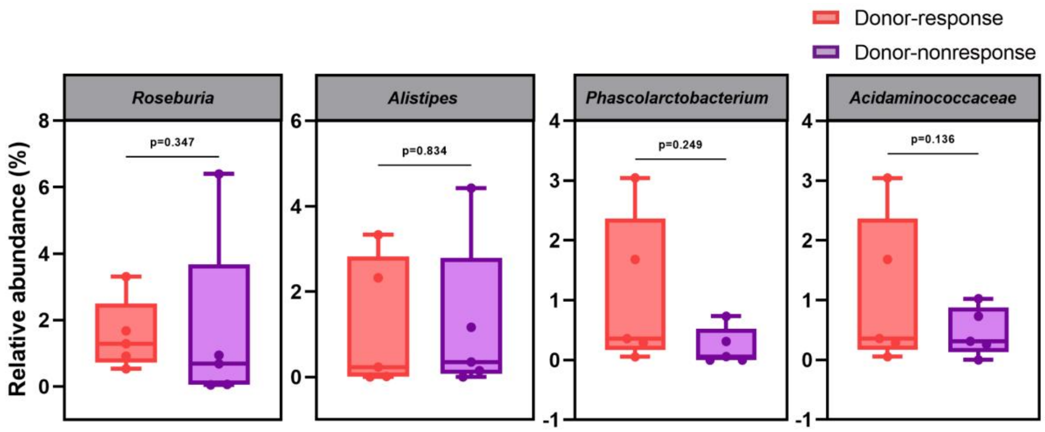

Fig. S5 Relative abundance of selected taxa in donors associated with responders and non-responders. The relative abundance of *Roseburia*, *Alistipes*, *Phascolarctobacterium,* and *Acidaminococcaceae* in donor stools paired with responders and non-responders is shown. The *P* values were generated by LEfSe (non-parametric Kruskal-Wallis tests).

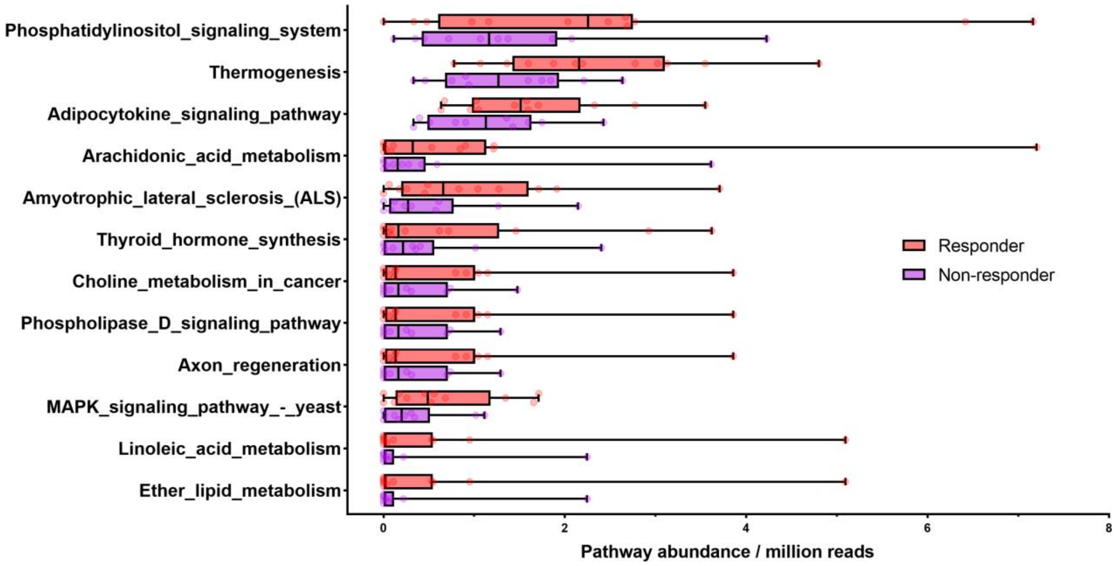

Fig. S6 Comparison of bacterial metabolic pathways between responders and non-responders at baseline. The relative abundance of metabolic pathways in responders and non-responders at baseline is presented. Statistical analyses were performed using independent-samples t-tests and Mann-Whitney U tests. *P* values were adjusted using the Benjamini-Hochberg FDR method. No significant differences were observed between the groups.

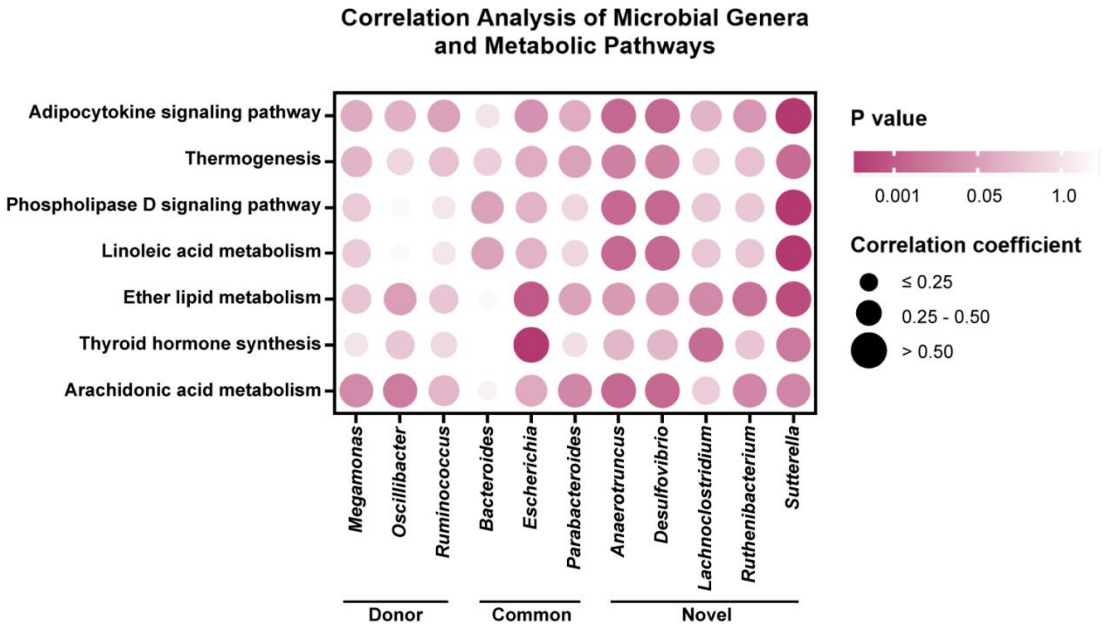

Fig. S7 Correlation between microbial genera and metabolic pathways in responders post-FMT. Pearson correlation was applied for normally distributed data, whereas Spearman correlation was used for non-normally distributed data. Only genera with statistically significant correlations with at least one of these metabolic pathways are presented.
